# Lutein plus Water Chestnut (*Trapa bispinosa* Roxb.) Extract Inhibits the Development of Cataracts and Induces Antioxidant Gene Expression in Lens Epithelial Cells

**DOI:** 10.1155/2020/9204620

**Published:** 2020-05-19

**Authors:** Hidetoshi Ishida, Teppei Shibata, Shinsuke Shibata, Yuki Tanaka, Hiroshi Sasaki, Eri Kubo

**Affiliations:** ^1^Department of Ophthalmology, Kanazawa Medical University, Ishikawa 9200293, Japan; ^2^Supplement Development & Promotion Group, Santen Pharmaceutical Co., Ltd., Tokyo 1030022, Japan

## Abstract

Age-related cataract (ARC) is the major cause of blindness worldwide. The most significant factors are the maximal exposure of the eye lens to environmental stressors, including oxidative and glycative load. The administration of antioxidant and antiglycative supplements may reduce the risk of cataract progression. In this study, the effects of lutein (LU) and water chestnut (*Trapa bispinosa* Roxb.) extract (TBE) on cataracts and the expression of antioxidant-related genes were assessed in Shumiya cataract rats (SCRs). LU+TBE or castor oil (COil) as a control was administered to 6- or 9-week-old cataractous SCRs and noncataractous SCRs via a feeding needle for 3 or 4 weeks. Five-week-old SCRs were provided *ad libitum* access to solid regular chow containing LU, TBE, LU+TBE, or the same chow without LU and/or TBE for 3 weeks. Lenses from all rats were then extracted and photographed. The right eyes of the rats were processed for histological observation, and the left eyes were used for total RNA extraction from lens epithelial cells (LEC). The mRNA levels of antioxidant proteins, peroxiredoxin 6, and catalase were examined using real-time quantitative polymerase chain reaction. Lens opacity appeared in all cataractous SCRs that began receiving LU+TBE at 9 weeks of age. However, compared to the COil group, lens opacity was decreased in the cataractous LU+TBE SCRs in all experiments. The mRNA expression levels of peroxiredoxin 6 and catalase in LECs of cataractous SCRs and cultured human LECs increased after the administration of LU+TBE. Collectively, our results highlight the anticataract and antioxidative effects of LT+TBE in SCRs. LT+TBE supplementation may, thus, be useful in delaying cataract progression.

## 1. Introduction

Age-related cataracts (ARC) are the leading cause of blindness worldwide and were responsible for 51% of the 39 million cases of blindness in 2010 [1]. Aging, oxidative stress, smoking, ultraviolet (UV) light, radiation, diabetes, and steroid use are among the risk factors for ARC [2, 3]. The lens of the human eye is susceptible to these stresses due to an accumulation of genetic changes in lens epithelial cells (LECs), as well as a lack of protein turnover which results in the aggregation of crystallin protein and an increase in insoluble protein levels [4]. *α*-Crystallin is a major water-soluble structural protein of the mammalian eye. This protein exhibits chaperone-like activity in the eye lens and plays a critical role in maintaining lens clarity [5]. Due to its long life, *α*-crystallin is susceptible to several posttranslational modifications such as oxidation, nonenzymatic glycation, deamidation, and the isomerization of protein-constituting amino acids during aging and diabetes in lenses. Such stresses can trigger LEC apoptosis, which might initiate reduction of the antistress protection in LECs and fiber cells [4, 6]. The most significant factor that induces ARC is oxidative stress. Oxidative stress induces the initiation and progression of cataracts, due to either the diminished expression and activity of natural antioxidants as a result of aging or the increased generation of reactive oxygen species (ROS) [6, 7]. Various antioxidant enzymes and proteins, such as catalase, peroxiredoxin 6, superoxide dismutase, glutathione peroxidase, and glutathione, have been reported to be present in the lens and to be involved in maintaining lenticular homeostasis [7–10]. It has been reported that the administration of antioxidants reduces the risk of cataract progression [6, 9, 11–14]. For example, lutein (LU) and zeaxanthin, which are naturally occurring carotenoids in the eye, have the potential to reduce cataract progression by absorbing short-wavelength visible light and quenching ROS [15, 16]. Although large-scale randomized trials have reported that both a multivitamin and LU have a cataract prevention effect, the evidence is not yet convincing [11, 17, 18].

Glycative stress is also a risk factor of age-related diseases and diabetic complication. Glycated proteins form advanced glycation end products (AGEs) through the formation of carbonyl compound-dominated intermediates [19]. Previous reports suggest that the glycation of lens proteins is involved in the pathogenic mechanism of cataract formation as a result of aging and diabetes [20]. The formation of various AGEs such as N*ε*-(carboxymethyl)lysine (CML), pentosidine, N*ε*-(carboxyethyl)lysine (CEL), pyrraline, and methylglyoxal hydroimidazolone 1 (MG-H1) in human lenses has been reported [21–23]. In a cataractous lens, AGEs such as CML and pyrraline are present at higher levels than in noncataractous lenses [23, 24]. AGEs induce irreversible conformational changes in *α*A-crystallin, leading to a loss of the protein's chaperoning ability, which then leads to protein aggregation and protein insolubility that scatters light and causes visual impairment by inducing lens opacity [22, 25]. Thus, drugs and supplements having antiglycative activity may be effective to prevent ARC and diabetic cataract.

Recently, considerable effort has sought to identify therapeutic agents, including those of phytochemical origin, that can promote naturally occurring cellular antioxidant and antiglycation defense systems. In this study, we focused on water chestnuts (*Trapa bispinosa* Roxb.) (TB), which has an antiglycative effect. TB are annual aquatic plants of the family Trapaceae and are widely used in ayurvedic medicine for their phytochemical and nonnutritional components, such as flavonoids [26]. A recent study demonstrated that TB extract (TBE) is rich in polyphenols (25% *w*/*w*), including gallic acid, ellagic acid, and eugeniin [12]. Furthermore, it has been reported that TBE inhibits the glycation-mediated crosslinking and carbohydration of *α*-crystallin *in vitro* [27, 28]. Thus, TBE may help to prevent the progression of ARC and diabetic cataracts by reducing AGE formation.

Here, we aimed to examine the effects of LU and/or TBE on the suppression of cataracts in Shumiya cataract rats (SCRs). In addition, we examined the expression of genes that encode the antioxidant peroxiredoxin 6 and catalase in the same rats, as well as in a human lens cell line, and thereby identified the anticataract effect of these phytochemical therapeutic agents.

## 2. Materials and Methods

### 2.1. Animals

All animal experiments were approved by the Committee of Animal Research at Kanazawa Medical University (Permission no. 2017-07) and were conducted in accordance with the U.S. National Institutes of Health Guide for the Care and Use of Laboratory Animals, the recommendations of the ARVO Statement for the Use of Animals in Ophthalmic and Vision Research, and the Institutional Guidelines for Laboratory Animals of Kanazawa Medical University. SCRs (SCR/Sscr: NBRP Rat No. 0823) were supplied by the National BioResource Project-Rat, Kyoto University (Kyoto, Japan). Rats were housed in a pathogen-free barrier facility (12 h light-dark cycle) and fed a diet of regular chow (Nosan Co., Ltd., Kanagawa, Japan).

**Figure 1:**
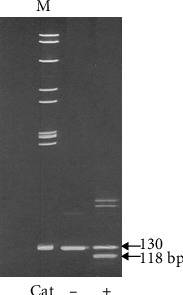
Detection of the mutation of lanosterol synthetase in Shumiya cataract rats (SCRs). Two bands were separated by electrophoresis, and the genotype of lanosterol synthetase in SCRs, cataract (Cat)+ or Cat−, was determined at 3–4 weeks of age.

We used 6- and 9-week-old SCRs. SCRs develop mild posterior and cortical cataracts at 8–9 weeks old, with mature cataracts appearing at 10–11 weeks of age [29]. Purified LU with castor oil (COil) (carbon monoxide) as a base (Koyo Mercantile Co., Tokyo, Japan) and peel extract of TB (TBE) (Hayashikane Sangyo Co., Yamaguchi, Japan) were administered to the SCRs. All rats were provided *ad libitum* access to regular or an experimental chow (Sankyo Labo Service, Tokyo). Four-week-old cataractous (Cat+) and noncataractous (Cat−) SCRs were distinguished by polymerase chain reaction (PCR) using genomic DNA from the rats' tails and 15% polyacrylamide gel electrophoresis (PAGE) to detect the mutation of lanosterol synthetase (Lss) (Figure 1). The sequences of primers used to detect the Lss mutation were as follows: 5′-GCACACTGGACTGTGGCTGG-3′ and 5′-GCCACAGCATTGTAGAGTCGCT-3′.

**Figure 2:**
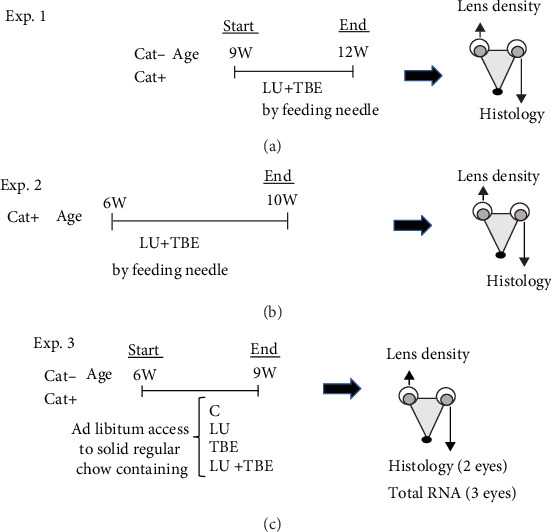
Experimental design for the administration of LU+TBE. Three types of experiments for the administration of LU and/or TBE were performed (a–c). LU: lutein; TBE: *Trapa bispinosa* extract.

We performed three experiments as shown in Figure 2. For the first experiment, a mixture of LU (2 mg/kg body weight (BW)) and TBE (20 mg/kg BW) dissolved in COil was administered to the experimental group of 9-week-old SCRs with or without cataracts (Cat+ or Cat-) via a feeding needle once a day for 3 weeks; the control group (C) received COil alone (*n* = 5 in each group) (Figure 2(a)). For the second experiment, the above mixture of LU and TBE dissolved in COil or COil alone was administered to 6-week-old Cat+ and Cat- SCRs via a feeding needle once a day for 4 weeks (*n* = 5 in each group) (Figure 2(b)). For the third experiment, 6-week-old Cat+ SCRs were provided *ad libitum* access to either solidified regular chow mixed with LU (3.25 mg/kg) and TBE (20 mg/kg) or the same chow without LU and TBE (C) for 3 weeks (*n* = 5 in each group) (Figure 2(c)). The volume of food in each rat's cage was measured every 3 days to calculate dietary intake.

At the end of the above-described experiments, the rats were sacrificed with CO_2_, and their lenses were carefully removed. Lenses from the right eyes of 5 rats in each group were photographed using a stereoscopic microscope with dark-field illumination (SMZ745T, Nikon Instech, Tokyo, Japan). The density of lens opacity was analyzed in each digital photograph of rat lenses with MultiGauge Software (Fujifilm, Tokyo, Japan). After being photographed, the right lenses were then processed for paraffin sectioning, stained with hematoxylin and eosin, and examined histologically. In the third experiment (Figure 2(c)), the LECs with lens capsules were removed from the left eyes of 3 rats to extract total RNA.

### 2.2. Cell Culture

Simian virus 40-transformed human lens epithelial cells (hLECs) (SRA01/04) (provided kindly by Dr. N. Ibaraki of the Ibaraki Eye Clinic, Tochigi, Japan) were cultured in Dulbecco's modified Eagle's medium (DMEM; Wako, Osaka, Japan), containing 20% fetal bovine serum (FBS; Sigma-Aldrich, St. Louis, MO, USA) to 80% confluence at 37°C in an air : CO_2_ (19 : 1) atmosphere for 24 h. For treatment with LU with or without TBE, cells were grown overnight on 35 mm plates and then incubated with 5 or 10 *μ*M LU dissolved in DMEM containing 0.1% dimethyl sulfoxide (DMSO) (Sigma-Aldrich) and 1% FBS (LU5 or LU10) and/or 50 *μ*g/mL TBE dissolved in DMEM containing 1% FBS. As a control (Cont), hLECs were cultured with DMEM containing 0.1% DMSO and 1% FBS. After an incubation period of 48 h, cells were washed and harvested for the preparation of cell extracts and total RNA. Three experiments were performed for each assay.

### 2.3. Real-Time Reverse Transcriptase-Quantitative PCR (RT-qPCR)

Total RNA was extracted from hLECs, as well as from the LECs with rat lens capsules of the excised left eyes of the rats with or without cataracts (Cat+ or Cat-) in the control group and the LU+TBE group in the third animal experiment with the use of an miRNeasy Micro Kit (Qiagen, Valencia, CA, USA) according to the manufacturer's instructions. To measure the mRNA expression of the rat or human peroxiredoxin 6 gene (*Prdx6* and *PRDX6*, respectively) and the rat or human catalase gene, we conducted a relative quantification of mRNA using an Applied Biosystems® 7300 system (Thermo Fisher Scientific Japan, Tokyo, Japan). PCR amplification was performed using TaqMan Universal Master Mix and a predeveloped rat peroxiredoxin 6 and catalase probe mix (Thermo Fisher Scientific Japan). The relative quantities of peroxiredoxin 6 and catalase mRNA were determined using the comparative Ct method and then normalized using a predeveloped TaqMan ribosomal RNA control reagent VIC probe as an endogenous control (Thermo Fisher Scientific Japan).

### 2.4. SDS-PAGE and Western Blotting

The LECs with rat lens capsules of the excised left eyes of the rats with or without cataracts (Cat+ or Cat-) were lysed in ice-cold radioimmune precipitation buffer, and SDS-PAGE was performed as described previously [30, 31]. After blocking with Odyssey blocking buffer (LI-COR, Lincoln, NE) for 30 min, the membranes were blotted with antiperoxiredoxin 6 antibody (Ab) (abcam, Cambridge, United Kingdom) and anticatalase Ab (Sigma-Aldrich), with antiglyceraldehyde-3-phosphate dehydrogenase (GAPDH) Ab as loading control and diluted to 1 : 2,000 in a solution of Odyssey blocking buffer and Tris-buffered saline, followed by incubation with secondary antibodies. The membranes were then scanned with an Odyssey infrared (IR) scanner. Quantification was performed using MultiGauge® software (Fujifilm, Tokyo, Japan).

### 2.5. Statistical Analysis

The results are reported as the mean ± standard deviation and were statistically analyzed using ANOVA with Fisher's test.

## 3. Results

### 3.1. Effects of LU+TBE on Cataract Progression in SCRs

Exp. 1: oral administration of LU+TBE via a feeding needle for 3 weeks beginning at the age of 9 weeks.

**Figure 3:**
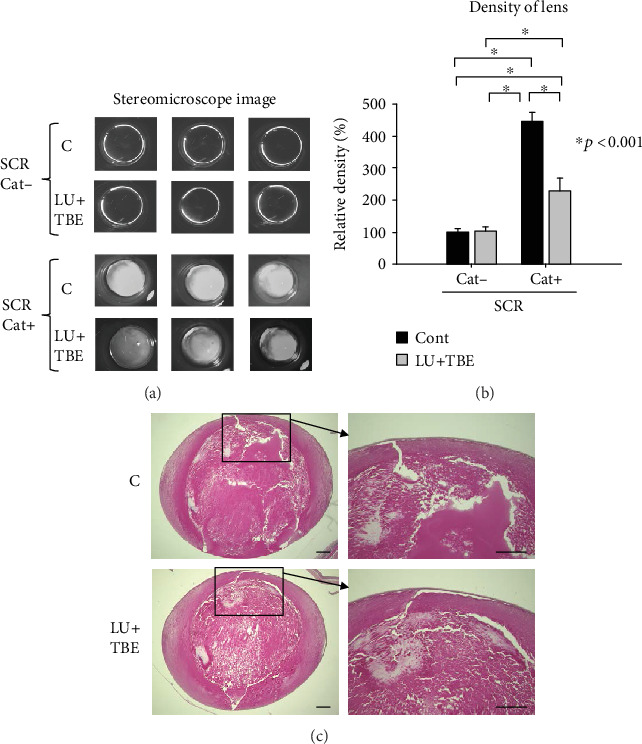
Observation of lens opacity in 12-week-old SCRs administered LU+TBE by a feeding needle from the age of 9 weeks. (a) Stereoscopic observation of representative lenses. (b) The relative density of lenses. Data are expressed as the mean + standard deviation (^∗^*p* < 0.001). (c) Histological analysis of representative lens. Bar = 80 *μ*m. SCR: Shumiya cataract rats; LU: lutein; TBE: *Trapa bispinosa* extract.

No lens opacity was observed in the Cat− SCRs following LU+TBE administration (Figure 3(a)). Lens opacity was apparent in the Cat+ SCRs (Figure 3(a)). The LU+TBE group showed slightly decreased lens opacity and significantly decreased lens density compared to the C group (Figure 3(b)). Histological analysis revealed that lens fiber damage in the nuclear portion was present in both the LU+TBE and control groups (Figure 3(c)), but the area of normal cortical fibers was slightly larger in the LU+TBE group compared to that in the control. It is possible that 9 weeks of age is too late for LU+TBE administration to be effective.

Exp. 2: oral administration of LU+TBE via a feeding needle for 3 weeks beginning at the age of 6 weeks.

**Figure 4:**
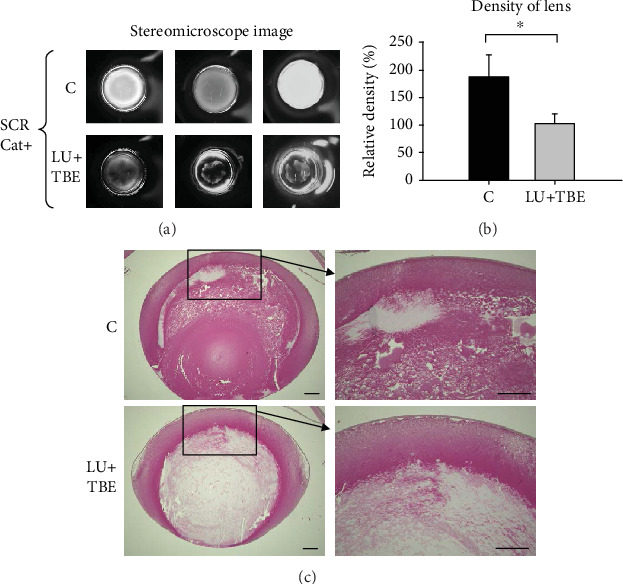
Observation of lens opacity in 10-week-old SCRs administered LU+TBE through a feeding needle from the age of 6 weeks. (a) Stereoscopic observation of representative lenses. (b) The density of lenses. Data are expressed as the mean + standard deviation (^∗^*p* < 0.03). (c) Histological analysis of representative lens. Bar = 80 *μ*m. SCR: Shumiya cataract rats; LU: lutein; TBE: *Trapa bispinosa* extract.

Compared to the control group, the progression of lens opacity was suppressed and lens density was significantly decreased in the LU+TBE group among the Cat+ SCRs (Figures 4(a) and 4(b)). The degree of fiber damage in the nucleus and posterior portion of the lens differed between the control and LU+TBE groups (Figure 4(c)).

Exp. 3: *ad libitum* chow with or without LU+TBE or TBE only for 3 weeks from the age of 6 weeks.

**Figure 5:**
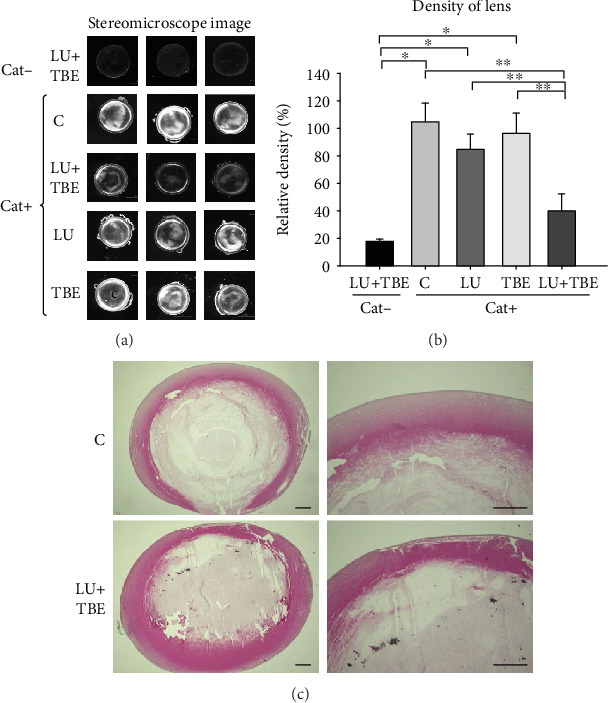
Observation of lens opacity in 9-week-old SCRs after ad libitum access to chow with or without LU+TBE or TBE alone from the age of 6 weeks. (a) Stereoscopic observation of representative lenses. (b) The density of lenses. Data are expressed as the mean + standard deviation (^∗^*p* < 0.0001, ^∗∗^*p* < 0.005). (c) Histological analysis of representative lens. Bar = 80 *μ*m. SCR: Shumiya cataract rats; LU: lutein; TBE: *Trapa bispinosa* extract.

According to the results of Experiment 2, 10-week-old SCRs exhibited mature cataracts. Therefore, in Exp. 3, LU±TBE was administered to a 6-week-old rat for 3 weeks. Lens opacity was observed in the Cat+ SCRs (Figure 5(a)). In the LU+TBE Cat+ SCR group, lens density was significantly decreased when compared to the control, LU-alone, and TBE-alone groups (Figure 5(b)). In the Cat+ group, lens nucleus depression, fiber swelling, and liquefaction were slightly suppressed in the LU+TBE rats compared to that in the control (Figure 5(c)).

### 3.2. Expression of Peroxiredoxin 6 and Catalase in the LECs of 9-Week-Old SCRs Administered LU±TBE and in hLECs Treated with/without LU±TBE

**Figure 6:**
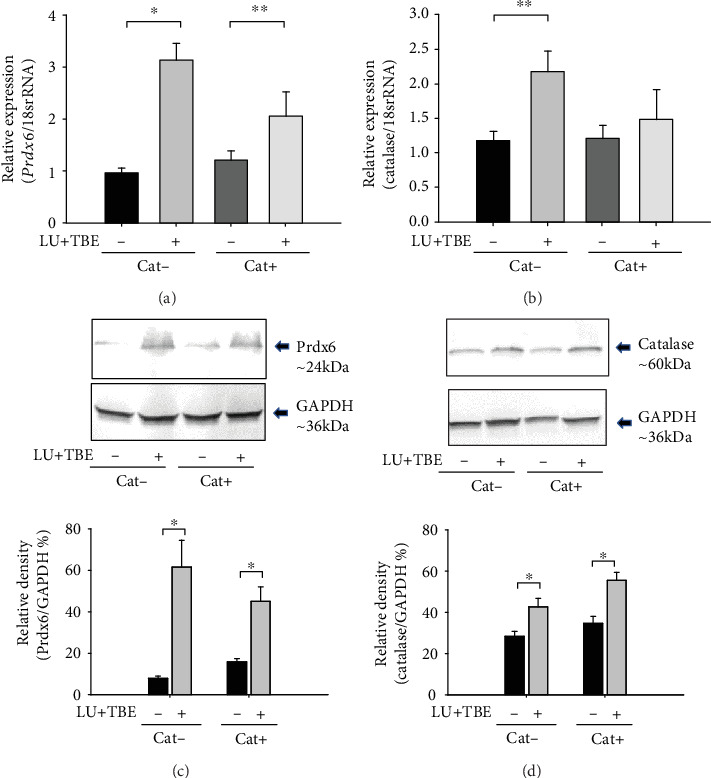
Expression of peroxiredoxin 6 (*Prdx6*) and catalase in LECs from 9-week-old SCRs after access ad libitum chow with or without LU and TBE from the age of 6 weeks. (a) The expression of *Prdx6* mRNA. (b) The expression of catalase mRNA. (c) The expression of Prdx6 protein. (d) The expression of catalase protein. Data are expressed as the mean + standard deviation. ^∗^*p* < 0.001, ^∗∗^*p* < 0.02. LEC: lens epithelial cells; SCR: Shumiya cataract rats; LU: lutein; TBE: *Trapa bispinosa* extract.

**Figure 7:**
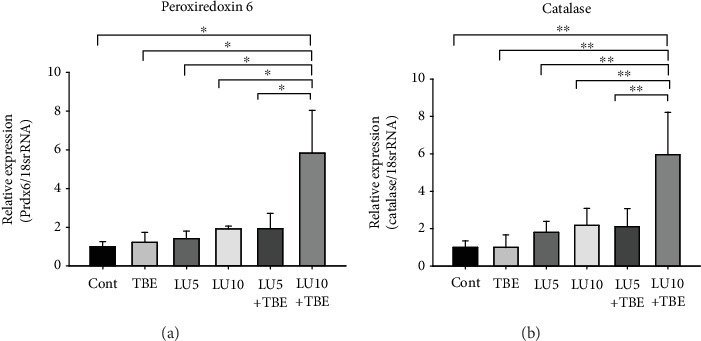
Expression of *Prdx6* and catalase mRNA in hLECs treated with/without LU and TBE. (a) The expression of *Prdx6* (^∗^*p* < 0.025). (b) The expression of catalase mRNA (^∗∗^*p* < 0.015). Data are expressed as the mean + standard deviation. hLEC: human lens epithelial cells; LU: lutein; TBE: *Trapa bispinosa* extract.

The expression of peroxiredoxin 6 and catalase mRNA and protein was significantly increased in the LECs of the LU+TBE group (Figures 6(a)–6(d); ^∗^*p* < 0.001, ^∗∗^*p* < 0.02 vs. Cat− and Cat+ control). The expression of peroxiredoxin 6 mRNA was also significantly increased in the LU10+TBE hLECs compared to that in the control (Figure 7(a); ^∗^*p* < 0.025 vs. all other groups). The expression of catalase mRNA was significantly increased in the LU10+TBE hLECs (Figure 7(b); ^∗^*p* < 0.015 vs. all other group).

## 4. Discussion

In this study, we assessed the combined effects of LU and TBE on cataract formation in SCRs. Our observations indicate that LU+TBE may delay the progression of lens opacity in these rats. For the rats administered LU+TBE beginning at 6 weeks of age, lens opacity was more suppressed than that in the group that only began to receive LU+TBE at 9 weeks of age. Starting administration of the LU and TBE supplements before the onset of cataracts is more effective in suppressing cataracts than starting these supplements after the development of lens opacity. In the group to which either LU or TBE was administered alone, lens opacity was not significantly suppressed. LU and zeaxanthin are the only carotenoids present in human serum, retina (macula), and lenses [32, 33] that are not synthesized *in vivo* [34]. LU and zeaxanthin can filter out UV and blue light, reducing ROS in the lens, and may thereby decrease light- or other stress-induced oxidative damage to the lens [35, 36], indicating that LU and zeaxanthin may prevent cataracts. Large-scale epidemiological studies suggest that LU and zeaxanthin reduce the risk of developing nuclear cataracts in ARC [17, 37]. Moreover, several human meta-analyses indicate that the incidence of nuclear cataracts is significantly decreased in individuals with high serum concentrations of LU and zeaxanthin [11, 38]. TBE inhibits the production of AGEs such as CML in *α*-crystallin [28]. Supplementation of LU or LU in combination with TBE delays the onset of cataracts in streptozocin-induced diabetic rats [12, 39]. These results suggest that cataracts in SCR may be inhibited by the antioxidative and antiglycative effects of combined administration of LU and TBE.

There is no appropriate animal model for age-related cataracts on which to study the effect of anticataract drugs. In this study, we used SCRs as a cataract rat model. SCRs are a hereditary cataractous strain in which 66.7% of animals develop lens opacity [29]. Mature cataracts appear in 10–11-week-old SCRs, and cortical and posterior opacity appeared in the 9-week-old SCRs in our study. Lens opacity progresses rapidly in SCRs after the age of 9 weeks. Cataract onset in SCR lenses is related to a combination of mutant alleles in the genes that encode lanosterol synthase and farnesyl-diphosphate farnesyltransferase 1, which decrease cholesterol levels [40]. It is thought that the continuous occurrence of apoptosis or cell death in LECs and poorly differentiated lens fiber cells at the bow area of the lens forms the cortical cataract formation due to cholesterol deficiency in SCRs [40]. Zhao et al. reported that lanosterol suppresses and redissolves crystallin aggregates and reduces lens opacity in rabbits and dogs. The research and development of lanosterol as an anticataract drug are in progress [41]. Moreover, Makley et al. reported that a sterol chaperone compound suppressed the aggregation of crystallin [42]. Therefore, the SCR model is attracting worldwide attention as a cataract animal model for the study of anticataract drugs.

We observed that the level of ROS in SCR lenses was elevated and that both the mRNA and the protein expression of peroxiredoxin 6 decreased [9]. The LEC layer is the initial site of attack by oxidative stress, followed by the involvement of the lens fibers, leading to cortical cataract [10]. We speculated that higher generation of ROS may be induced by the reduced expression of antioxidant genes such as peroxiredoxin 6 and in SCRs and may lead to apoptosis of LECs [9]. We previously studied the anticataract effect of peroxiredoxin 6 in SCRs [9]. The peroxiredoxin 6 expressed in murine and human lenses decreases with age [31, 43]. The progression of lens opacity in SCRs is delayed by the administration of a peroxiredoxin 6-recombinant protein with a TAT signal domain [9]. It has also been demonstrated that aminoguanidine (an inhibitor of advanced glycation) strongly inhibits the development of lens opacification in SCRs [44]. Thus, antioxidative and antiglycative therapy using LU and TBE was effective to delay the progression of lens opacity.

Here, we examined whether the inhibition of oxidative and glycative stress by administration of LU and TBE affects the expression of internal antioxidant genes such as peroxiredoxin 6 and catalase in SCR lenses. We demonstrated that these antioxidative genes are significantly upregulated in SCR LECs (with or without cataracts) and hLECs following LU and TBE treatment. We found that LU and TBE can induce endogenous antioxidant gene expression in rat and human LECs and may thereby accelerate the reduction of intracellular ROS. Thus, LU and TBE may not only have antioxidant and antiglycation activity themselves but may also induce endogenous antioxidant genes and delay the onset of cataracts.

## 5. Conclusions

The results of our present study demonstrated that the simultaneous administration of LU and TBE can delay the onset of cataracts in SCRs and induce antioxidant gene expression in LECs. Simultaneous administration of LU and TBE may delay ARC by reducing oxidative stress and glycative stress in aged lenses. To achieve efficacious anticataract activity, it is necessary to reduce not only oxidative stress but also glycative stress.

## Data Availability

All data generated or analyzed during this study are included in this published article. More details are available from the corresponding author upon reasonable request.
